# Elaboration of Prussian Blue Analogue/Silica Nanocomposites: Towards Tailor-Made Nano-Scale Electronic Devices

**DOI:** 10.3390/ma5030385

**Published:** 2012-03-05

**Authors:** Giulia Fornasieri, Merwen Aouadi, Emilie Delahaye, Patricia Beaunier, Dominique Durand, Eric Rivière, Pierre-Antoine Albouy, François Brisset, Anne Bleuzen

**Affiliations:** 1 Institut de Chimie Moléculaire et des Matériaux d’Orsay—Equipe de Chimie Inorganique, UMR 8182, Université Paris-Sud, Orsay 91405, France; E-Mails: giulia.fornasieri@u-psud.fr (G.F.); merwen.aouadi@u-psud.fr (M.A.); emilie.delahaye@u-psud.fr (E.D.); eric.riviere@u-psud.fr (E.R.); 2 Laboratoire de Réactivité de Surface, UMR 7197, CNRS, Université Pierre et Marie Curie-Paris VI, Ivry 94200, France; E-Mail: patricia.beaunier@upmc.fr; 3 Institut Biochimie et Biophysique Moléculaire et Cellulaire, UMR 8619, Université Paris-Sud, Orsay 91405, France; E-Mail: dominique.durand@u-psud.fr; 4 Laboratoire de Physique des Solides, UMR 8502, Université Paris-Sud, Orsay 91405, France; E-Mail: pierre-antoine.albouy@u-psud.fr; 5 Institut de Chimie Moléculaire et des Matériaux d’Orsay—Service de Microscopie Electronique, UMR 8182, Université Paris-Sud, Orsay 91405, France; E-Mail: francois.brisset@u-psud.fr

**Keywords:** Prussian blue analogue, mesoporous silica, confined precipitation, photomagnetism, sol-gel

## Abstract

The research of new molecular materials able to replace classical solid materials in electronics has attracted growing attention over the past decade. Among these compounds photoswitchable Prussian blue analogues (PBA) are particularly interesting for the elaboration of new optical memories. However these coordination polymers are generally synthesised as insoluble powders that cannot be integrated into a real device. Hence their successful integration into real applications depends on an additional processing step. Nanostructured oxides elaborated by sol-gel chemistry combined with surfactant micelle templating can be used as nanoreactors to confine PBA precipitation and organize the functional nano-objects in the three dimensions of space. In this work we present the elaboration of different CoFe PBA/silica nanocomposites. Our synthetic procedure fully controls the synthesis of PBA in the porosity of the silica matrix from the insertion of the precursors up to the formation of the photomagnetic compound. We present results on systems from the simplest to the most elaborate: from disordered xerogels to ordered nanostructured films passing through mesoporous monoliths.

## 1. Introduction

Prussian blue analogues (PBA) are cyano-bridged coordination polymers that have attracted growing attention over the past decades because of their multiple electronic properties (magnetic, photomagnetic, electrochemical). Among these compounds photomagnetic PBA [1], *i.e.*, compounds of which the magnetic properties change following an irradiation, are particularly interesting for the development of new electronic devices such as optical memories. At present, several works report on the study of the properties of these photoswitchable Prussian blue analogues constituted by different bimetallic pairs (CoFe [1,2,3,4,5,6], MnFe [7,8,9], FeCr [10,11,12]), and exhibiting electronic structure changes induced by different external stimuli (light [1,13,14,15], temperature [3,16,17], pressure [12,18,19,20,21]). The chemical and physical properties of these molecular materials depend on their size, shape and spatial organization. Hence the control of these parameters and the study of their effects on the properties is a prerequisite for future applications. 

Up to now several works have reported on the control of the size of PBA particles. Coprecipitation in aqueous solutions with controlled synthetic conditions can lead to nanosized particles [22]. Moreover several synthetic confining media have been considered: reverse micelle [23,24,25], periphery of direct miniemulsion [26,27], polymers [28,29,30,31] or biopolymers [32,33], ionic liquids [34], alumina membranes [35,36], silica xerogels [37] or mesostructured silicas [38,39,40]. 

Nevertheless the elaboration of real devices based on the photoswitchable properties of these compounds requires a processing step leading to a macroscopic material constituted by ordered arrays of nanoparticles. In order to assemble and organize these functional objects, solid matrices that exhibit well-defined pore size, pore shape and pore organization, and that can also be deposited onto various substrates, are particularly suited. Nanostructured silica, elaborated by sol-gel chemistry combined with surfactant templating, combine all these capabilities. We therefore decided to use these silica matrices as hard templates to process photomagnetic PBAs. 

However, only a few works report the elaboration of Prussian blue analogues within the porosity of mesoporous silica. The precipitation of Prussian blue analogue involves substitution of the water molecules of a hexa-aqua metallic complex [M(H_2_O)_6_]^z+^ by [M′(CN)_6_]^q−^ anions. This reaction is so fast that the introduction of both reactive precursors within the nanoreactors generally leads to uncontrolled precipitation and pore obstruction. Thus, our first attempts to precipitate CoFe PBA within the porosity of ordered mesoporous silica powders systematically ended with PBA external precipitation. In order to overcome this major limitation, we have developed a step-by-step approach in order to take full advantage of the exceptional processing flexibility inherent in nanostructured oxide sol-gel chemistry to process PBAs.

**Figure 1:**
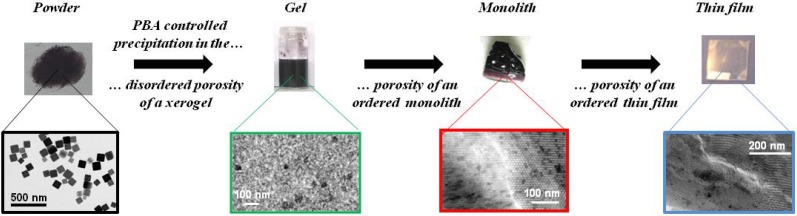
Illustration of the step-by-step approach for Prussian blue analogues (PBAs) processing.

In this work we report the step-by-step approach, which led us to progressively control the precipitation of CoFe Prussian blue analogue first in the disordered porosity of xerogels, then in the porosity of ordered mesoporous silica monoliths and finally in the ordered mesoporosity of thin films (Figure 1). The reported strategy can be easily adapted to the elaboration of various Prussian blue analogues or derivatives.

The simultaneous control of the structure of the nanocomposite at different scales (nano-, micro- and millimetric) necessitates a good understanding of the reactivity of all the components of the system. The keystone of the entire process is cobalt cation chemistry in a complex medium containing several interactive species: acid or base catalysed silica sol, cobalt cations and Fe(CN)_6_ entities and structuring agents. Understanding of the reactivity of the cobalt(II) species allowed us to elaborate an original synthetic strategy towards PBA-silica nanocomposites with full control of the morphology, the chemical composition (and the spatial organization for the ordered silica) of CoFe PBA nanoparticles.

## 2. Results and Discussion

### 2.1. Controlled Precipitation of CoFe PBA within the Disordered Porosity of Silica (Xero)Gels

CoFe PBA/silica nanocomposites are obtained by controlled precipitation of CoFe PBA nanoparticles in the pores of silica xerogel [41].

The elaboration of the nanocomposite involves two distinct condensation processes: precipitation of CoFe PBA and polymerization of silica. Precipitation of CoFe PBA involves substitution of the water molecules of the hexa-aquacobalt(II) complex [Co(H_2_O)_6_]^2+^ by [Fe(CN)_6_]^3−^ anions [5]. Given the lability of the hexa-aquacobalt(II) complex (the water-exchange rate constant for [Co(H_2_O)_6_]^2+^ at 298 K is k = 3.18 × 10^6^·s^−1^ [42]), the precipitation of CoFe PBA can be considered as instantaneous compared to the polymerization reactions of the silica matrix. The silica gel is obtained by hydrolysis-condensation of tetraethyl orthosilicate (TEOS); such reactions are very slow at neutral pH but can be catalysed by addition of an acid or a base. The acid catalysis is less effective but produces a polymeric gel with nanometer-sized pores. On the other hand the basic polymerization is faster but generates a particulate gel with larger pores that are less effective to confine PBA precipitation. The two-step acid-base catalysed process is therefore a good compromise to obtain a faster polymerization rate and a smaller porosity [43]. The silica polymerization rate is nevertheless still much slower than CoFe PBA precipitation, therefore the direct incorporation of CoFe PBA precursors in a silica host most often results in phase segregation between the silica gel and the CoFe PBA precipitate. Confined precipitation of the CoFe PBA in the pores of the silica matrix can be obtained by the simultaneous control of both condensation processes.

**Figure 2:**
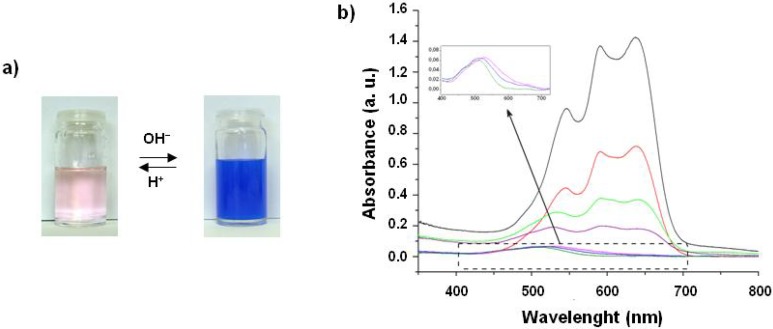
(**a**) Photos of the Co(II)-containing silica sols in an acidic (left) and basic (right) medium (sols and gels have the same appearance); (**b**) UV-Visible spectra of the Co(II)-containing silica gels obtained after addition of NaOH aqueous solutions with different concentrations 0 M (dark green), 0.1 M (blue), 0.2 M (violet), 0.3 M (purple), 0.4 M (green), 0.5 M (red), 1 M (black)).

The CoFe PBA precursors were first separately introduced into the silica matrix prepared by the two-step acid-base catalysed process and then the gel and xerogel phases were characterised. Addition of basic potassium hexacyanoferrate(III) solution to the prehydrolyzed silica sol gave an opaque yellow gel, due to the precipitation of K_3_[Fe(CN)_6_], which is only partially soluble in the medium. Addition of Co(II) nitrate to the prehydrolyzed acidic sol (pH ≈ 2) resulted in a pink coloration of the sol (Figure 2a). Subsequent addition of a 2 M aqueous NaOH solution to the cobalt(II)-containing sol (pH ≈ 9) was accompanied by an instantaneous color change of the sol from pink to deep blue (Figure 2a), followed by gelation of the sol. The spectrum of the pink sol (Figure 2b) shows a band in the visible range assigned to the ^4^T_1g_(F)→^4^T_1g_(P) transition (515 nm) which is the absorption signature of the octahedral [Co(H_2_O)_6_]^2+^ complex. The spectrum of the blue sol (Figure 2b) shows a strong absorption in the visible range and a multiple band (585 nm) assigned to the ^4^A_2_→^4^T_1_(P) transition of a Co(II) ion in a tetrahedral environment. This spectrum can be attributed to a Co(II) complex similar to [Co(OH)_4_]^2−^ that is obtained by hydroxylation of the hexa-aquacobalt(II) complex in strongly basic medium. The low electrophilicity of the cobalt atoms in the tetrahedral species and their homogenous dispersion in the silica matrix prevent condensation reactions leading to the corresponding cobalt hydroxide and/or oxide phases. It is important to note that the formation of this tetrahedral Co(II) complex upon increasing the pH is reversible. Thus, acidification of the blue gel makes it turn light pink again with an absorption spectrum characteristic of the [Co(H_2_O)_6_]^2+^ complex. 

**Figure 3:**
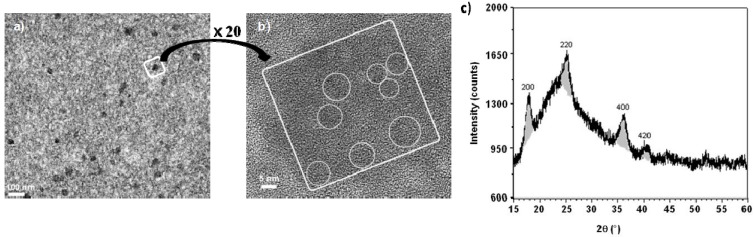
(**a**) Transmission electron microscopy (TEM) image of the RbCoFe PBA/silica xerogel nanocomposite (microtomed sample); (**b**) High resolution transmission electron microscopy (HRTEM) image of one RbCoFe PBA/nanoparticle (microtomed sample). The white square surrounds one PBA aggregate and the circles inside on figure b indicate the cristallographic domains; (**c**) XRD pattern of the RbCoFe PBA/silica xerogel nanocomposite (Cu K_α1_ = 0.1540598 nm).

Addition of a solution of K_3_[Fe(CN)_6_] to the blue Co(II)-containing sol gives a green sol, which leads to a gel (or a xerogel). The green color suggests that the two species did not react together, as confirmed by the FTIR absorption band at 2116 cm^−1^ corresponding to the stretching vibration of a terminal CN^−^ anion bonded to Fe(III) ion. The tetrahedral hydroxylated Co(II) complex does not react with the CN^−^ nucleophilic species of the [Fe(CN)_6_]^3−^ complexes, probably because of the low electrophilicity of the Co(II) ion and the negative charge borne by the blue basic species. Hence it is possible to introduce the CoFe PBA precursors without precipitation of the corresponding PBA during silica polymerization. Once the silica network has formed, precipitation of PBA can be simply triggered by adding an acidic solution to the medium. Indeed, acidification of the green (xero)gel with an aqueous HNO_3_ solution transforms the basic tetrahedral Co(II) complex into the reactive cationic [Co(H_2_O)_6_]^2+^ complex. Actually the acidification of the green (xero)gel is accompanied by an instantaneous color change to reddish-brown, which is evidence of the precipitation of CoFe PBA in the (xero)gel porosity. The presence of a large excess of Rb^+^ cations in the HNO_3_ solution leads to the formation of a violet RbCoFe PBA, containing a significant amount of alkali-metal cations stabilizing the Co(III)-Fe(II) diamagnetic photoswitchable pairs [5]. The FTIR spectrum of the nanocomposite confirms the oxidation states of the metallic ions. Thus, in addition to the signals of the silica matrix, a broad absorption band, centered at 2125 cm^−1^ with a shoulder at lower energy, is present. This signal can be attributed to the envelopement of two contributions: the stretching vibration of the majority of bridging cyanides in the Co(III)-NC-Fe(II) environment together with some in the Co(II)-NC-Fe(II) environment. The X-ray powder diffraction pattern confirms the face centered structure typical of a Prussian blue analogue with a cell parameter value of de 9.97 ± 0.05 Å compatible with a majority of low spin Co(III) ions (Figure 3c) [5]. Elemental analysis of an as-synthesized nanocomposite gives the following chemical formula: Rb_2.3_Co_4_[Fe(CN)_6_]_3.4_(SiO_2_)_842_(H_2_O)_374_.

The nanocomposite is constituted of PBA nanoparticles homogenously dispersed in the matrix, as shown by transmission electron microscopy (TEM) images of microtomed samples. These nanoparticles are about 40 ± 12 nm in diameter (Figure 3a). They are constituted by the assembly of several crystallographic domains, each of about 8 nm, as supported by high resolution TEM image (Figure 3b).

The Prussian blue analogue of formula Rb_2_Co_4_[Fe(CN)_6_]_3.3_·11H_2_O freely precipitated in aqueous solution, named bulk RbCoFe PBA, shows a remarkable photomagnetic effect. Irradiation of the compound with visible light at low temperature results in an electronic transfer that transforms the Co(III)-Fe(II) diamagnetic pairs into Co(II)-Fe(III) magnetic pairs with a net increase of the magnetization of the material. This state is metastable and heating above the relaxation temperature (110 K) leads back to the fundamental state [5]. 

**Figure 4:**
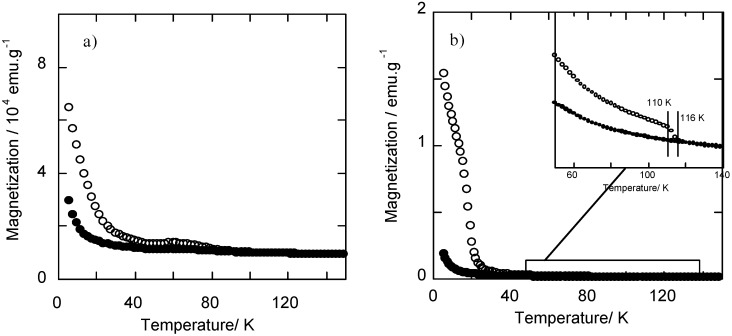
Magnetization curves for (**a**) the RbCoFe PBA/silica nanocomposite and (**b**) the bulk RbCoFe PBA before (●) and after (○) irradiation.

An analogous photomagnetic effect was also verified for the nanoparticles precipitated in the silica matrix (Figure 4a). Thus, irradiation of RbCoFe PBA/silica nanocomposite with a red laser at 10 K results in an increase in magnetization, which can be erased by heating up to 100 K.

The bulk RbCoFe PBA, shows a ferrimagnetic behaviour, with a T_c_ of 21 K, after irradiation (Figure 4b). This magnetic ordering temperature is not detectable for the RbCoFe PBA/silica nanocomposite, probably because of the nanometric size of the crystallographic domains.

### 2.2. Controlled Precipitation of CoFe PBA within the Porosity of Nanostructured Silica Monoliths

The confined precipitation of the Prussian blue analogue in the pores of a mesostructured silica monolith yields nanocomposites containing PBA nanosized single crystals (~5 nm) with a tridimensional organization [44].

The elaboration process consists in the synthesis of a Co(II)-containing mesoporous silica monolith followed by the impregnation with an acidic hexacyanoferrate(III) solution to precipitate the CoFe Prussian blue analogue inside the pores. The use of a silica monolith, with a small external surface/volume surface ratio, instead of micrometric powder effectively confines the precipitation of PBA inside the pores, without diffusion of precursors to the surroundings and formation of PBA on the silica surface or in the impregnation medium. Moreover the chemistry of mesoporous silica monoliths is very versatile, monoliths with different mesostructures and different cobalt contents can be synthesized allowing the elaboration of nanocomposites containing CoFe PBA particles with different morphologies (particles, wires, lamellae or tridimensional replica) [45]. 

We present here the results on monoliths with a 2D-hexagonal porosity but this method can be extended to other tridimensional organizations (3D-cubic, lamellar) [46].

**Figure 5:**
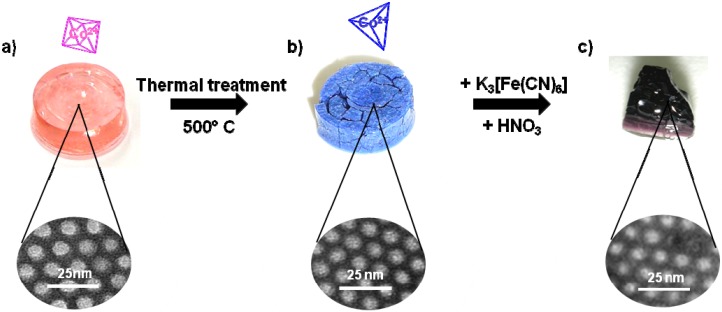
Photographs of (**a**) the Co(II)-containing silica copolymer monolith, (**b**) the Co(II)-containing silica mesoporous monolith obtained after thermal treatment, and (**c**) the ordered CoFe PBA/silica nanocomposite after impregnation with an acidic solution of K_3_[Fe(CN)_6_].

**Figure 6:**
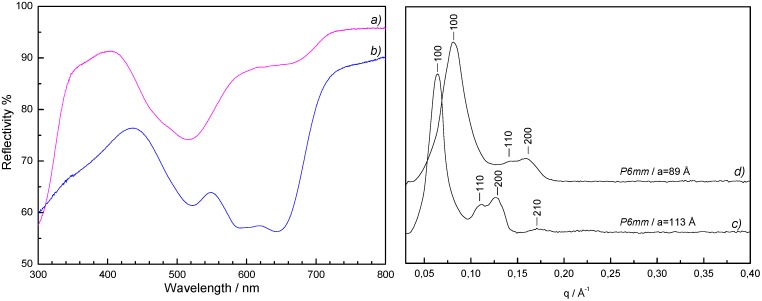
On the left UV-visible spectra of the Co(II)-containing silica monoliths (**a**) before and (**b**) after thermal treatment at 500 °C. On the right, small angle XRD patterns of the Co(II)-containing silica monoliths (**c**) before and (**d**) after thermal treatment at 500 °C.

Mesoporous Co(II)-containing silica monoliths were fabricated by adapting the synthetic procedure reported by El Safty that makes use of instantly preformed liquid crystalline phases in bulk lyotropic systems to obtain ordered mesoporous sol-gel silica monoliths [47]. The synthetic procedure begins with the solubilisation of the triblock copolymer Pluronic P123 (EO_20_PO_70_EO_20_) with tetramethyl orthosilicate (TMOS), followed by the addition of an aqueous acidified solution of cobalt(II) nitrate to quickly achieve the liquid crystal phase and promote the polymerization of TMOS around the 2D-hexagonal lyotropic assembly of the copolymer. The product of silica polymerization is a Co(II)-containing pink glassy silica-copolymer monolith (Figure 5a) with a 2D-hexagonal P6mm nanostructuration (cell parameter value a = 113 Å). The UV-visible spectrum (Figure 6a) is similar to the spectrum of the pink sol reported in §2.1 and is the signature of the octahedral [Co(H_2_O)_6_]^2+^ complex.

Elimination of the templating agent is a necessary step to liberate the porosity before impregnation. To preserve the integrity of the monolith, ethanol extraction is generally preferred to remove the surfactant. In this case, the treatment with ethanol vapours in a Soxhlet extractor led to the bleaching of the monolith and a pink Pluronic P123 and cobalt(II) nitrate ethanolic extracting solution. Hence extraction is not suitable to liberate the porosity and obtain a Co(II)-containing mesoporous silica monolith. Nevertheless, this experience suggests that cobalt(II) ions are not incorporated in the silica walls but are in interaction with the polyether polymer and would be accessible after elimination of the templating agent by thermal degradation.

The structuring agent was then removed by calcination at 500 °C in air. During thermal treatment the monolith cracked into some sub-centimeter size fragments and their colour changed from pink to blue (Figure 5b). Small angle X-ray diffraction patterns showed a shift towards higher q values indicating a lattice contraction of the condensed silica phase (cell parameter value a = 89 Å) accompanied by a slight enlargement of the peaks due to a slight loss of organization but preservation of the 2D-hexagonal organization (Figure 6, curves c and d). The UV-visible spectrum (Figure 6, curve b) of the blue calcined monolith is typical of a tetrahedral Co(II) ion. 

The modification of the coordination sphere geometry of the Co(II) cation is due to the thermohydrolysis of the hexa-aquacobalt(II) complex giving a tetrahedral monomer [48] as already reported for silica gel after alkalinization. As already seen for the basic gel (Section 2.1) the tetrahedral complex is unreactive towards ferricyanide but the reactive [Co(H_2_O)_6_]^2+^ species can be recovered by impregnation with an acidic solution. The impregnation of the blue monolith with ferricyanide acidic solution leads to the precipitation of CoFe Prussian blue analogue inside the pores (Figure 5c).

**Figure 7:**
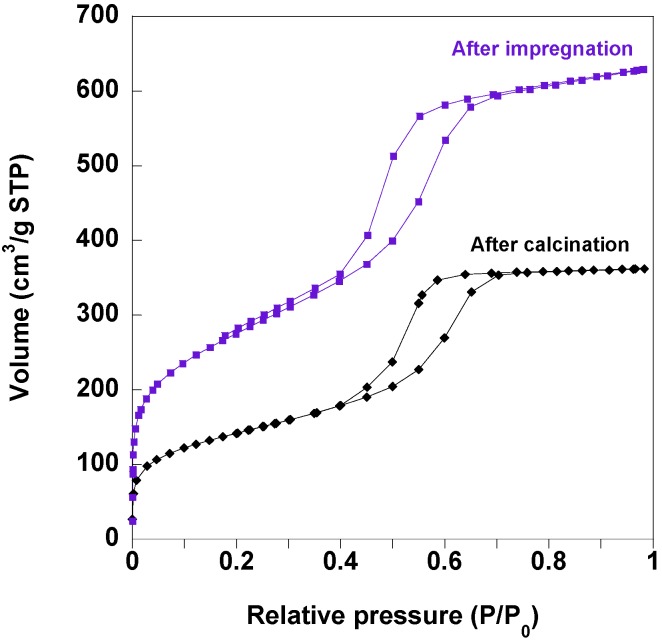
N_2_ adsorption/desorption isotherms of the monoliths containing 2% wt of Co(II) ions after thermal treatment at 500 °C (black curve) and after impregnation in an acidic solution of K_3_[Fe(CN)_6_] (violet curve).

Porosity of calcined monoliths before and after impregnation was studied by nitrogen adsorption-desorption isotherms. The calcined monoliths containing 2% of Co(II) ions exhibit an isotherm of type IV which is associated with capillary condensation and desorption taking place in mesopores (Figure 7). This compound shows clear type H_1_ hysteresis loops that are related to uniform open-ended cylindrical mesopores. As expected, the amount of adsorbed nitrogen, *i.e.*, the porous volume, as well as the BET (Brunauer-Emmett-Teller) surface is reduced after impregnation with ferricyanide solution and precipitation of CoFe PBA particles inside the pores (Figure 7). The total pore volume calculated at P/P_0_ = 0.98 is of 0.97 cm^3^∙g^−1^ and 0.56 cm^3^∙g^−1^ before and after the impregnation respectively, which indicates partial plugging of the pores by the precipitated PBA particles. No change in the isotherm type is observed after impregnation proving that the geometry of the pores is preserved.

**Figure 8:**
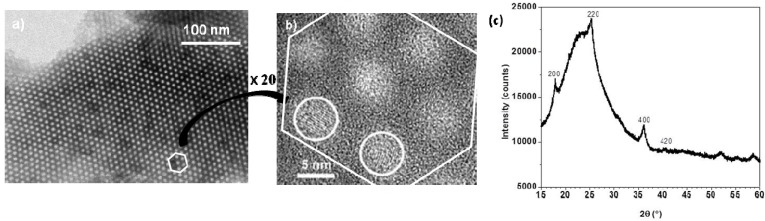
(**a**) TEM micrograph of the RbCoFe PBA/silica monolith nanocomposite (transverse section of microtomed sample); (**b**) HRTEM micrograph of the same nanocomposite. White circles surround the RbCoFe PBA single crystalline nanoparticles; (**c**) XRD pattern of the RbCoFe PBA/silica monolith nanocomposite (Cu K_α1_ = 0.1540598 nm).

Transmission electron microscopy (TEM) images of the nanocomposites show PBA nanoparticles as dark regions inside the pores (Figure 8a). High resolution transmission electron microscopy (HRTEM) images (Figure 8b) show in the 2D-hexagonal mesoporosity of the silica monolith single-crystal nanoparticles with averaged measured lattice planes spacing (0.363 ± 0.004 nm, 0.300 ± 0.004 nm, 0.250 ± 0.004 nm and 0.220 ± 0.004 nm) consistent with the (220), (222), (400) and (422) *d*-spacings of RbCoFe PBA. The PBA precipitation is confirmed by the XRD pattern of the nanocomposite (Figure 8c) showing the diffraction lines characteristic of PBAs.

**Figure 9:**
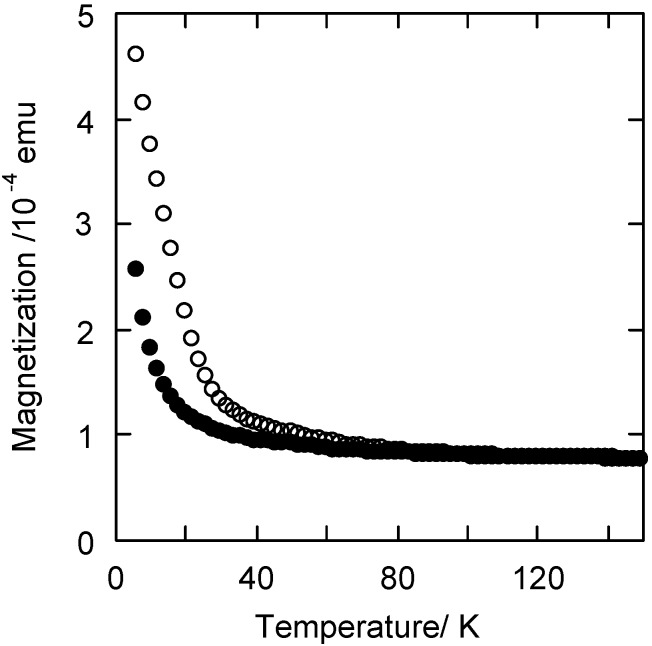
Temperature dependence of the magnetization before (●) and after (○) irradiation (T = 10 K λ = 642 nm, P = 15 mW·cm^−2^) of RbCoFe PBA/silica nanocomposite.

The magnetization curves of the RbCoFe PBA/silica nanocomposite before and after irradiation with a red laser at 10 K show that the nanocomposite exhibits a photomagnetic effect (Figure 9). Its photomagnetic properties are different from those of bulk RbCoFe PBA of the same stoichiometry (Figure 3b). The irradiation of RbCoFe PBA produces an increase in magnetization due to a photo-induced electron transfer from Fe(II) to Co(III) transforming the diamagnetic Co(III)-Fe(II) pairs into the magnetic Co(II)-Fe(III) ones. At 5 K, the magnetization is multiplied by 10 in the bulk compound after irradiation whereas it is only multiplied by 2 in the nanocomposite. This difference is due to particle size reduction in the nanocomposite accompanied by a loss of magnetic ordering temperature in the photo-induced metastable state but, as for the xerogel, the electron transfer is totally preserved even in particles of 5 nm in diameter.

### 2.3. Controlled Precipitation of CoFe PBA within the Porosity of Nanostructured Silica Thin Films

The synthetic strategy described in Section 2.2 has been adapted to the elaboration of silica nanostructured thin films containing CoFe PBA nanoparticles.

The initial Co(II)-containing mesoporous silica film is prepared by spin-coating of a silica sol, containing Co(II) cations and the block copolymer Pluronic P123, on glass substrates. The synthetic procedure to prepare the sol had to be adapted from the one giving Co(II)-containing silica monoliths. Actually, the high viscosity of the silica sols, prepared for the elaboration of the monoliths, prevents the formation of a homogenous film. In order to obtain a more liquid sol to produce nanostructured silica films through the evaporation-induced self-assembly (EISA) approach, the formulation and synthetic conditions presented in §2.2 were modified by adding methanol and ageing for one hour. 

The self-assembly leading to the mesostructuration of the film occurs during spin-coating through the EISA approach. Both templating agents and inorganic precursors are cooperatively self-assembled at the surface of the substrate by evaporation of the solvent that initially solubilised all the components. The films deposited on the glass substrate appear continuous and show a good optical quality.

**Figure 10:**
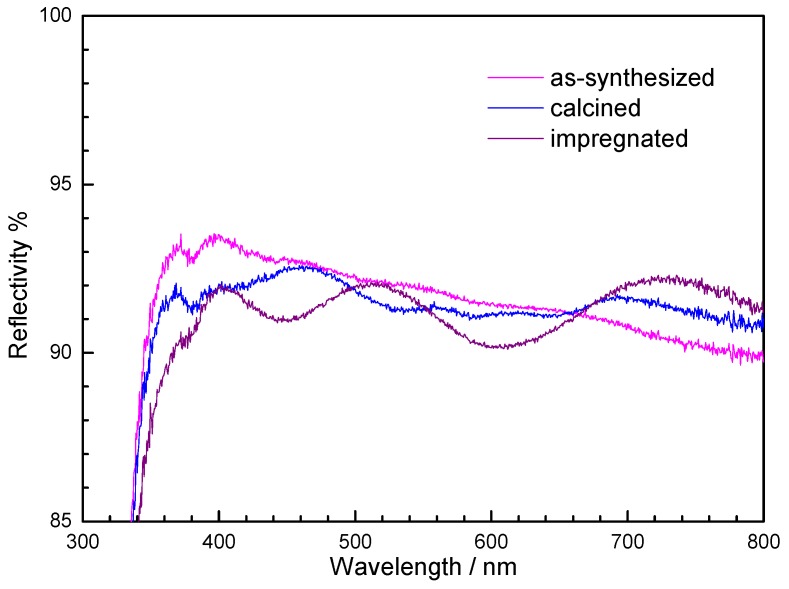
UV-visible spectra of the Co(II)-containing silica films: (**a**) as-synthesized; (**b**) after thermal treatment at 500 °C; (**c**) after impregnation with the ferricyanide acidic solution.

As presented in Section 2.2 the templating agent was eliminated to liberate the porosity by a thermal treatment at 500 °C in air. After calcination the film appears still crack-free and adheres well to the support. The appearance of a light blue colour suggests a geometry change of the Co(II) coordination polyhedron from octahedral to tetrahedral by thermally activated hydrolysis of the hexa-aquacobalt(II) complex. The reflectance UV-visible spectra of the as-synthesized film and the film after thermal treatment are shown in Figure 10. The spectrum of the as-synthesized film shows absorption bands of very weak intensity. The hardly detectable band around 520 nm is the signature of the octahedral [Co(H_2_O)_6_]^2+^ complex. The spectrum of the calcined film shows a more pronounced multiple band centred around 585 nm, confirming the presence of tetrahedral [Co(OH)_4_]^2−^-like complex. 

**Figure 11:**
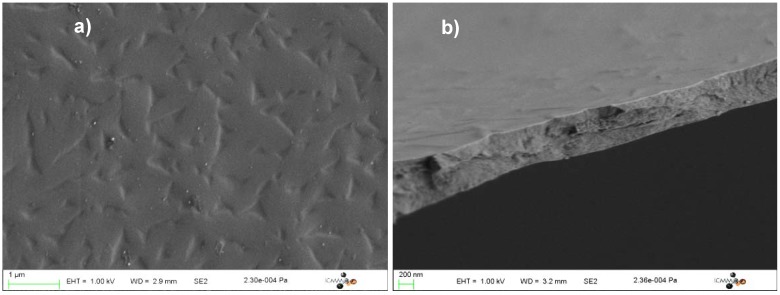
Scanning electron microscopy (SEM) micrographs of the calcined Co(II)-containing silica films: (**a**) Top-view; (**b**) Side-view.

The scanning electron microscopy (SEM) images of the calcined Co(II)-containing silica films are shown in Figure 11. The calcined films are crack-free at the microscopic scale and uniform over hundreds of micrometres. Their thickness lies within the 500–1000 nm range. No particle is detectable on the surface. 

**Figure 12:**
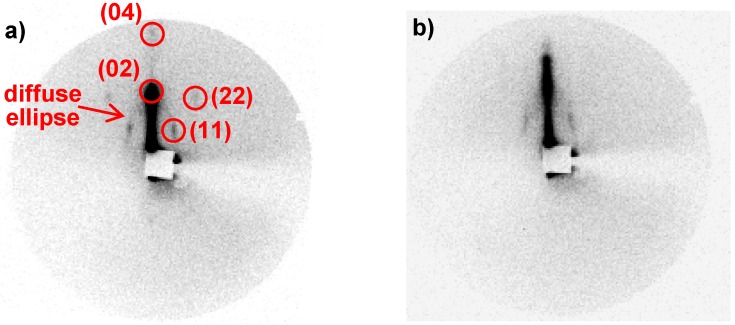
2D XRD diffraction patterns obtained at grazing incidence of the Co(II)-containing silica film: (**a**) as synthesized; (**b**) after thermal treatment at 500 °C.

The films were analysed by 2D XRD at grazing incidence to study the nanostructuration. The patterns (Figure 12) show a diffuse ellipse and some well-defined diffraction spots. These patterns are consistent with a 2D centered rectangular lattice (*c2m*) due to a contraction of the original 2D hexagonal lattice (*p6m*) [49]. The diffraction spots positioned on the ellipse are due to well-aligned domains that have their *c* axis preferentially aligned along the surface plane while the ellipsoidal continuous diffraction is attributed to random orientations of porosity domains. This random orientation of porosity domains can originate either from the deposition method or from the roughness of the substrate.

**Figure 13:**
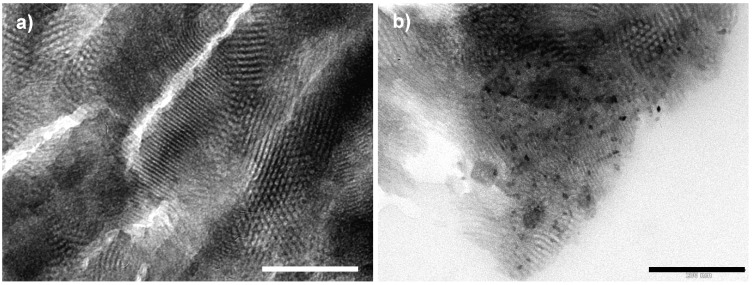
(**a**) TEM image of the thermally treated Co(II)-containing silica film. (microtomed sample); (**b**) TEM image of RbCoFe PBA/silica film nanocomposite (microtomed sample). Scale bar is 200 nm.

TEM analysis was performed on calcined films before (Figure 13a) and after impregnation with the ferricyanide acidic solution (Figure 13b). Before impregnation, the micrograph confirms X-ray diffraction showing different contracted 2D-hexagonal domains with several orientations. 

As reported in Section 2.2, the precipitation of RbCoFe PBA nanoparticles inside the porosity was obtained by simple impregnation of the film with an acidic solution containing potassium ferricyanide and rubidium nitrate. The precipitation of PBA particles was proved first by UV-visible spectroscopy (Figure 11c) which showed the disappearance of the absorption of the [Co(OH)_4_]^2−^-like species and the apparition of new ones assigned to charge transfer bands usually observed in RbCoFe PBA. The TEM image of the film after impregnation (Figure 13b) shows that the nanostructuration is preserved after calcination and that the PBA particles formed during the impregnation step are exclusively located inside the porosity of the nanostructured film.

## 3. Experimental Section

### 3.1. Materials Characterization

Elemental analyses of Si, Co, Fe, C, N, H were performed at the analysis facility of the CNRS in Solaize.

UV-Visible spectra were recorded at room temperature, over the 200–800 nm range, with a Varian Cary 5000 spectrometer equipped for solid samples with an internal diffusion reflectance accessory DRA 2500 (spectra were recorded with a mean resolution of 0.5 nm and a sampling rate of 300 nm·min^−1^).

Fourier Transformed-Infrared (FT-IR) spectra were collected in the transmission mode on a Perkin Elmer Spectrum100 spectrometer.

Small angle X-ray diffraction measurements were performed on a Nanostar laboratory instrument from Bruker-AXS (Karlsruhe, Germany). X-Rays (wavelength 1.54 Å) from a rotating anode generator Microstar with microfocus (100 × 100 μm^2^) go through double multilayer element optics in a Montel arrangement followed by three circular pinholes of 0.75, 0.4 and 1 mm diameter respectively. For each sample we obtain the scattered intensity I(q) as a function of the momentum transfer q (q = (4πsinθ)/λ) over the q-range [0.012 Å−1–0.5 Å−1]. 

Two-dimensional (2D) X-ray scattering analyses are performed with a rotating anode X-ray generator (copper anode operated at 50 kV, 30 mA; small focus) equipped with a doubly curved graphite monochromator, delivering an intense beam with a limited resolution (maximal accessible d spacing: ≈8 nm). A vacuum pipe reduces the air scattering. The scattering is recorded on photostimulable imaging plates. The exposure time ranges from 14 min to 15 h.

Nitrogen adsorption–desorption isotherms were measured at liquid nitrogen temperature using a Belsorp-Mini (BelJapan Inc., Osaka, Japan), in standard operating mode. Prior to analysis, all samples were degassed for seven hours at 120° C under vacuum (P < 2 × 10^−3^ torr). The single-point total pore volume was estimated *ca.* P/P_0_ = 0.98. The pore diameters were calculated according to the Barrett-Joyner–Halenda (BJH) model on the desorption branch. The Brunauer-Emmett-Teller (BET) specific surface area was obtained in the 0.05–0.30 relative pressure range.

Transmission electron microscopy (TEM) images were recorded with a JEOL JEM 100CXII electron microscope operating at an acceleration voltage of 100 kV. Materials were analysed after ultramicrotomy. For high resolution microscopy (HRTEM) images, we used a JEOL JEM 2010 equipped with LaB_6_ filament and operating at 200 kV. The images were collected with a 4008 × 2672 pixels CCD camera (Gatan Orius SC1000). The chemical analyses were obtained by a selected energy-dispersive X-ray spectroscopy (EDS) microanalyzer (PGT-IMIX PC) mounted on the JEM 2010. 

Scanning Electron Microscopy was realized on a Zeiss Supra 55 VP. SEM images were acquired using a field emission gun (FEG) SEM working at very low voltage (1 kV) in order to avoid the deposit of a conductive layer. The advantage of this solution is to be sure that the observation shows the surface structure of the material and in any case the structure of any conductive layer. The working distance is small but this does not affect the observation.

Magnetization measurements were performed in a SQUID magnetometer (Quantum Devices MPMS5) equipped with an optical fiber made of multiwire silica. The fiber was connected to a laser diode (642 nm) with P = 15 mW. The magnetization data for each sample were obtained as follows. In a first step, a paste constituted of a small amount of RbCoFe PBA/silica nanocomposites and nujol was prepared and spread on a paper support of 5.5 mm in diameter. The magnetization of the samples was measured over the 5–200 K temperature range in the heating mode, using a 5000 Oe applied field. Finally, each compound was irradiated for 15 min at 10 K, and the magnetization was measured for a second time as a function of temperature.

### 3.2. Synthesis of RbCoFe PBA/Silica Disordered Nanocomposites

Silica gel is prepared by a two-step hydrolysis process. The first step consists of mixing at 60 °C tetraethyl orthosilicate (8 g, 38.4 mmol), ethanol (5.4 g), water (0.7 g mmol, 38.4 mmol) and nitric acid 37% (1 μL). After 1.5 h, cobalt(II) nitrate hexahydrate (47 mg, 0.16 mmol) is solubilised in the sol at room temperature. While the pink sol is still being stirred, a KOH aqueous solution (2 M) is added in two fractions for the second step. First 0.87 mL of KOH 2 M, then 0.87 mL of KOH 2 M containing 0.16 mmol of potassium hexacyanoferrate(III) are added. The green sol gels in 30 seconds. The resulting gel is aged in a sealed vial for one week and dried at room temperature. 

The green xerogel is dispersed in 30 mL of a rubidium acidic solution (2.42 mmol of RbNO_3_ in HNO_3_ 0.6%): the rubidium CoFe Prussian blue analogue precipitates in the silica matrix giving a violet nanocomposite. The final product is washed three times with water to eliminate unreacted ions and recovered by centrifugation.

### 3.3. Synthesis of RbCoFe PBA/Silica Nanostructured Monoliths

For a typical synthesis, 2.4 g of Pluronic P123 was added to 4 g of tetramethyl orthosilicate (TMOS) in a 30 ml polypropylene vial and the solution was shaken in a water bath at 55 °C until the polymer had completely dissolved. After cooling to room temperature, 2 ml of an aqueous acidic cobalt(II) nitrate hexahydrate solution [pH = 1.4, 80 mg Co(NO_3_)_2_∙6H_2_O] was quickly added to the stirred silicate-block copolymer clear solution and was distributed in four vials. Then each vial was sealed tightly and the homogenous pink mixture was transferred to a water thermostated bath at 23 °C to be aged for one hour without stirring. After removing the vial lid, the resulting viscous sol gelled in 6 h giving a coloured translucent gel, the ageing process was continued one week more to give a homogenous pink glassy Co^2+^-containing silica-copolymer monolith. The polymerisation of silica was completed by a treatment at 80 °C overnight.

The CoFe PBA-silica nanocomposite is obtained by precipitation of CoFe PBA inside the pores of the silica monolith. The first step is the thermal treatment of the synthesised Co^2+^-containing silica-copolymer monolith to liberate the porosity. The treatment consists of heating from room temperature to 250 °C in 6 h, an isothermal plateau over 30 minutes, followed by a second heating to 500 °C in 2 h and an isothermal plateau over 6 h. Afterwards the resulting blue monolith is impregnated with a 0.1 M potassium hexacyanoferrate(III) aqueous solution containing nitric acid (1.13 M) and rubidium nitrate (0.5 M) over two min. A colour change from blue to violet indicating the precipitation of the CoFe PBA nanoparticles is readily observed. The final product was washed three times with water to eliminate residual ions and dried at room temperature.

### 3.4. Synthesis of RbCoFe PBA/Silica Nanostructured Films

For a typical synthesis, 2.4 g of Pluronic P123 was solubilised in 4 g of TMOS in a water bath at 55 °C. After cooling to room temperature, 10 mL of methanol was added. Then, 2 mL of an aqueous acidic cobalt(II) nitrate hexahydrate solution [pH = 1.4, 80 mg Co(NO_3_)_2_∙6H_2_O] was quickly added to the stirred silicate-block copolymer clear solution. The resulting sol was aged for one hour at room temperature. The sol was then spread evenly on a glass substrate and spun coated at a spinning rate of 4,000 rpm for 90 seconds. 

The films were dried one night at room temperature and then calcined in air at 500 °C for 6 h to remove the surfactant. Thus, transparent thin films were formed on the substrates. 

Finally, the resulting light blue film was immersed in a 0.1 M potassium hexacyanoferrate(III) aqueous solution containing nitric acid (1.13 M) and rubidium nitrate (0.5 M) over two min. The film was thoroughly rinsed three times with water to eliminate residual ions and dried at room temperature.

## 4. Conclusions

Coordination and molecular compounds offer promising perspectives for the elaboration of nanomaterials able to reproduce on the nanometric scale classical functions used in the electronic industry. However, a processing step is mandatory in order to integrate them into real applications. Nanostructured oxides elaborated by sol-gel chemistry combined with surfactant micelle templating with the inherent exceptional processing flexibility constitute appealing hard templates to shape and position functional objects in space. This approach necessitates the combination of sol-gel and coordination chemistries, which is, *a priori*, not straightforward.

In this work, we propose an original step-by-step approach, which enables us to take advantage progressively of the processing flexibility inherent in the sol-gel silica chemistry to elaborate Prussian blue analogue-based nanomaterials showing photo-switchable properties. The versatile chemistry of Co(II) ions enabled us, by controlling the polymerisation processes of the inorganic networks of silica and Prussian blue analogues, to elaborate PBA/silica gels and xerogels nanocomposites. Then, the well-known interactions between transition metal ions and the block copolymer, containing ether functions, combined with the versatile chemistry of the Co species, were used to adapt the strategy to the elaboration of PBA/ordered mesoporous silica monoliths nanocomposites. Finally, the combination of the EISA approach to the previous procedure afforded PBA/nanostructured silica film composites.

These nanocomposites are very well suited to study of the effect of the processing step (particles size, shape, organization within an external field) on the electronic properties of the functional nano-objects and they offer promising perspectives towards the elaboration of tailor made nanodevices.
